# The *Drosophila melanogaster* Metabolic Response against Parasitic Nematode Infection Is Mediated by TGF-β Signaling

**DOI:** 10.3390/microorganisms8070971

**Published:** 2020-06-29

**Authors:** Yaprak Ozakman, Trishya Pagadala, Dhaivat Raval, Ioannis Eleftherianos

**Affiliations:** Infection and Innate Immunity Laboratory, Department of Biological Sciences, Institute for Biomedical Sciences, The George Washington University, Washington, DC 20052, USA; yozakman@gwmail.gwu.edu (Y.O.); trishya.pagadala@gmail.com (T.P.); draval7@gwmail.gwu.edu (D.R.)

**Keywords:** *D. melanogaster*, *Heterorhabditis*, metabolism, parasitism, TGF-β signaling

## Abstract

The nematode *Heterorhabditis bacteriophora*, its mutualistic bacterium *Photorhabdus luminescens*, and the fruit fly *Drosophila melanogaster* establish a unique system to study the basis of infection in relation to host metabolism. Our previous results indicate that the Transforming Growth Factor β (TGF-β) signaling pathway participates in the *D. melanogaster* metabolic response against nematode parasitism. However, our understanding of whether the presence of *Photorhabdus* bacteria in *Heterorhabditis* nematodes affects the metabolic state of *D. melanogaster* during infection is limited. Here, we investigated the involvement of TGF-β signaling branches, Activin and Bone Morphogenetic Protein (BMP), in the *D. melanogaster* metabolic response against axenic (lacking bacteria) or symbiotic (containing bacteria) *H. bacteriophora* infection. We show that BMP signaling mediates lipid metabolism against axenic or symbiotic *H. bacteriophora* and alters the size of fat body lipid droplets against symbiotic nematode infection. Also, following symbiotic *H. bacteriophora* infection, Activin signaling modulates sugar metabolism. Our results indicate that Activin and BMP signaling interact with the *D. melanogaster* metabolic response to *H. bacteriophora* infection regardless of the presence or absence of *Photorhabdus*. These findings provide evidence for the role of TGF-β signaling in host metabolism, which could lead to the development of novel treatments for parasitic diseases.

## 1. Introduction

The innate immune response against microbial infection is often associated with the metabolic state of the host [1,2,3,4,5,6]. In turn, the metabolic state of an organism is a critical determinant of a functional immune system. Several components of the immune system modulate metabolism, and together these processes are paramount for tissue and organismal homeostasis [7].

The nematode parasite *Heterorhabditis bacteriophora* together with its symbiotic bacteria *Photorhabdus luminescens* and the fruit fly *Drosophila melanogaster* constitute a well-suited model to dissect the molecular basis of infection in relation to the host metabolic response. *Heterorhabditis* nematodes infect various insects at their infective juvenile (IJ) stage. Upon entry, the nematodes expel their symbiotic bacteria into the insect hemolymph, where the bacteria secrete toxins and virulence factors to suppress the insect immune system [8,9]. The bacteria also serve as an essential nutrient source required for nematode growth and proliferation. When nematode numbers reach high density and resources are depleted, the IJs which are colonized by the bacteria, emerge from the insect cadaver to search for a new host [10].

In addition to its pivotal role in several developmental processes such as axis formation, body patterning, and morphogenesis, the Transforming Growth Factor-β (TGF-β) signaling pathway modulates anti-nematode immunity in *D. melanogaster* [6,11,12,13,14,15]. Components of the TGF-ß signaling pathway are expressed in immunologically, and metabolically active tissues in both vertebrates and invertebrates, and their roles in regulating immunity and metabolism have been gaining attention [16,17]. Similar to vertebrates, the TGF-ß pathway in *D. melanogaster* is composed of two signaling branches named Activin and Bone Morphogenetic Protein (BMP). The TGF-ß signaling pathway is initiated by the binding of an extracellular ligand to a transmembrane receptor complex of serine/threonine kinases [16,18]. The Activin branch is activated by Activin-β (Actβ), Dawdle (Daw) and Myoglianin (Myo) ligands, and the BMP branch is activated by Decapentaplegic (Dpp), Glass-bottom boat (Gbb) and Screw (Scw) [19]. Activation of the receptor leads to phosphorylation of downstream transcription factors that regulate the expression of target genes [20].

Previous studies have shown that extracellular ligands Daw and Dpp are involved in the immune response of *D. melanogaster* adult flies against parasitic nematode infection [14,15]. In addition, recent work has demonstrated that the TGF-ß signaling Activin and BMP branches participate in the *D. melanogaster* metabolic response against the nematode parasite *H. gerrardi* [6]. Both signaling branches regulate lipid metabolism, and the Activin pathway only interferes with glycogen metabolism upon *H. gerrardi* infection. However, our understanding of whether *Heterorhabditis* nematodes are capable of altering the metabolic state of *D. melanogaster* without any contribution from their associated bacterial symbionts remains currently unknown. Therefore, it is essential to separate the two symbiotic partners, nematodes and bacteria, and study the metabolic capacity of the insect host to infection with axenic (lacking *Photorhabdus* bacteria) or symbiotic (containing *Photorhabdus* bacteria) nematodes independently.

In order to elucidate the role and regulation of TGF-β signaling branches in the *D. melanogaster* metabolic response against infection with axenic or symbiotic *H. bacteriophora* nematodes, we used *D. melanogaster* larvae carrying loss-of-function mutations in *daw* and *dpp* genes coding for extracellular ligands in the Activin and BMP signaling branches, respectively. We measured carbohydrate, lipid and ATP levels, and determined the size of lipid droplets in the fat body of these mutants upon infection with axenic or symbiotic nematodes. Results obtained from this study provide novel insight into the role of TGF-ß signaling in host metabolism in the context of nematode infection and thus may lead to the identification of new strategies for treating parasitic infectious diseases.

## 2. Materials and Methods 

### 2.1. Fly Stocks

Fly stocks were maintained on *D. melanogaster* medium (Meidi Laboratories) supplemented with baker’s yeast (Carolina Biological Supply, Burlington, NC, USA) at 25 °C, and a 12:12-h light:dark photoperiodic cycle. Flies carrying a spontaneous dpp^s1^ mutation (strain 397, Bloomington, IL, USA) and a P-bac insertion Pbac{XP}daw05680 (strain d05680, Exelixis, Boston, MA, USA) were used in this study, as previously described [6]. Fly line *w*^1118^ (strain 3605, Bloomington, IL, USA) was used as background control. Late second to early third instar larvae were used for all experiments.

### 2.2. Nematode Stocks

Infective juveniles (IJs) of *H. bacteriophora* strain TT01 were amplified through the fifth to sixth instar larvae of the wax moth *Galleria mellonella* using the water trap technique [21]. Axenic *H. bacteriophora* nematodes lacking their symbiotic *P. luminescens* bacteria were generated by a previously established technique [22]. Prior to use, axenic nematodes were surface-sterilized in 10% bleach solution and rinsed five times with water to remove the bleach residue. All nematodes were used within one to four weeks after collection.

### 2.3. Larval Infection

*D. melanogaster* larvae were infected with axenic or symbiotic *H. bacteriophora* IJs in microtiter 96-well plates, containing 100 μL of 1.25% agarose in each well. The suspension of IJs (100 nematodes in 10 μL of sterile distilled water) was added into each well of the microtiter plate followed by a transfer of a single larva. Sterile distilled water (10 μL) was used as the uninfected control. The plate was covered with a sealing film (USA Scientific, Ocala, FL, USA) and air holes were pierced for aeration. Plates were kept in dark at room temperature for 24 h. At the 24-h time point, infected and uninfected larvae, were collected and frozen at −80 °C, or immediately used in experiments. Each infection experiment was performed three times with biological duplicates that included larvae from different generations.

### 2.4. RNA Analysis

Extraction of total RNA from five to seven *D. melanogaster* larvae was performed using TRIzol™ reagent following the manufacturer’s instructions. Reverse transcription and Quantitative RT-PCR (qRT-PCR) experiments were carried as previously described using gene specific primers for *Ribosomal protein L32* (*RpL32*), *Drosophila insulin-like peptide 6* (*Dilp6*), *Drosophila insulin-like peptide 3* (*Dilp3*), and *forkhead box-O* (*fOXO*) (Table 1) [6]. Each experiment was run in biological duplicates and repeated three times.

### 2.5. Measurement of Triglyceride, Trehalose, Glucose, and Glycogen Levels

*D. melanogaster* larvae were infected with axenic or symbiotic *H. bacteriophora* IJs and 24 h following infection, seven to nine larvae were collected and rinsed in 1× PBS. Larvae were then homogenized with a pellet pestle on ice in 100 μL of 1× PBS to determine glucose and glycogen levels, or in 100 μL Trehalase buffer (TB; 5 mM Tris pH 6.6, 137 mM NaCl, 2.7 mM KCl) to assess trehalose levels, or in 100 μL of PBST (1× PBS + 0.05% Tween 20) to measure triglyceride levels, using previously established protocols [23]. Protein quantification was determined by Pierce™ BCA Protein Assay Kit (Thermo Fisher Scientific; 23227, Waltham, MA, USA).

Triglyceride levels were determined by adding diluted samples (1:1 in PBS-Tween) and 200 μL of Infinity™ Triglycerides Liquid Stable Reagent (Thermo Fisher Scientific; TR22421) into a clear 96-well plate, which was incubated for 30 min at 37 °C. Following the incubation, absorbance was measured at 540 nm. The glycerol standard curve was used to calculate triglyceride concentrations.

To determine the levels of trehalose, samples were initially diluted 1:3 in TB and further diluted 1:1 in either TB or Trehalase Stock (TS; 3 μL of porcine trehalase in 1 mL of TB). Following an incubation at 37 °C for 24 h in a clear 96-well plate, hexokinase (Glucose Assay Reagent, Sigma-Aldrich; G3293, St. Louis, MO, USA) reagent (100 μL) was added to each well and the absorbance was measured at 340 nm. Trehalose levels were calculated from samples digested in TS by subtracting the amount of free glucose.

The levels of glucose and glycogen were measured by initially diluting the samples in 1:3 in PBS and then further diluting them to 1:1 in either amyloglucosidase stock solution (1.5 μL of amyloglucosidase in 1 mL of PBS, Sigma-Aldrich) or PBS. Following the incubation of diluted samples (30 μL) at 37 °C for 60 min in a clear 96-well plate, hexokinase reagent (100 μL) was added to each well and samples were incubated at room temperature for an additional 15 min. Absorbance was measured at 340 nm. Glucose standard curve was used to determine glucose levels. Glycogen levels were calculated by subtracting the absorbance of glucose from the absorbance of samples diluted with amyloglucosidase stock. 

The amounts of triglycerides, trehalose, glucose, and glycogen were calculated relative to the amount of proteins in each sample. All experiments were run in biological duplicates and repeated three times.

### 2.6. Measurement of Cholesterol and ATP Levels

*D. melanogaster* larvae were infected with axenic or symbiotic *H. bacteriophora* IJs and at 24 h post-infection, seven to nine larvae were collected and homogenized either in 100 μL of 1× reaction buffer provided by Amplex Red Cholesterol Assay Kit (Invitrogen; A12216, Carlsbad, CA, USA) for cholesterol quantification, or in ATP reaction mix (Molecular Probes ATP kit; A22066, Eugene, OR, USA) for measuring ATP levels. The amount of proteins was determined by Pierce™ BCA Protein Assay Kit (Thermo Fisher Scientific; 23227).

To determine cholesterol levels, 50 μL of samples were mixed with 90 μL of the reaction mix provided by the kit in black 96-well plates (Fisher Scientific; 509051574) and incubated for 30 min at 37 °C. Fluorescence was measured with excitation at 530 nm and emission at 590 nm.

To measure ATP levels, samples were initially diluted 1:10 and subsequently diluted further 1:75 in dilution buffer (25 mM of Tris, 100 μM of EDTA). Samples were then transferred to individual wells of a 96-well plate and the luciferase reaction mix (100 μL) was added to each well. Luminescence was measured immediately. Each experiment was performed in biological duplicates and repeated three times.

### 2.7. Lipid Droplet Staining

At 24 h following infection of *D. melanogaster* larvae with axenic or symbiotic *H. bacteriophora*, fat body tissues of 10 *D. melanogaster* larvae were dissected and fixed in 4% paraformaldehyde prepared in PBS at room temperature for 30 min. Tissues were then rinsed with PBS and incubated in the dark for 30 min in 0.05% of Nile red diluted 1:1000 in 1 mg/mL of methanol. ProLong™ Diamond AntiFade Mountant with DAPI (Life Technologies; P36962, Carlsbad, CA, USA) was used to mount tissues. Lipid droplets were visualized using a Zeiss LSM 510 confocal microscope. Quantification of lipid droplet size was assessed by measuring the area of the four largest lipid droplets per cell from four fat body cells using ImageJ software (National Institutes of Health, Bethesda, MD, USA).

### 2.8. Statistical Analysis

GraphPad Prism7 was used for data plotting and statistical analyses. Statistical analyses of all experimental results were performed using one-way analysis of variance (ANOVA).

## 3. Results

### 3.1. D. melanogaster daw Mutant Larvae Contain Lower Levels of Trehalose Upon Symbiotic H. bacteriophora Infection

**Figure 1:**
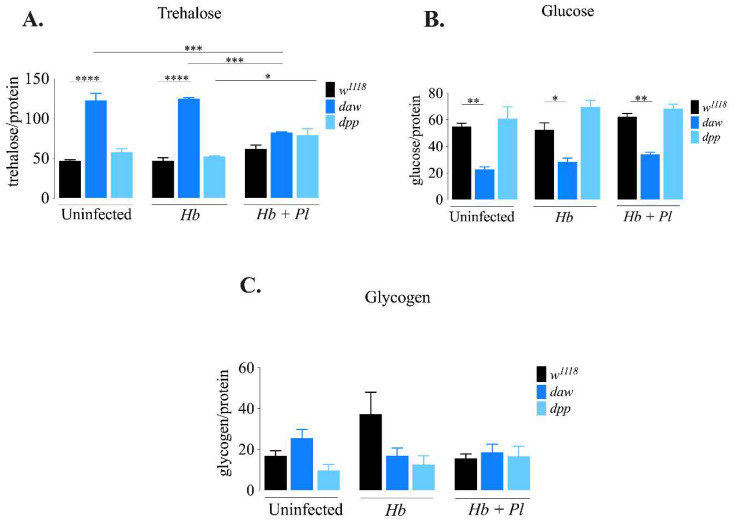
Carbohydrate metabolism in *Drosophila melanogaster* TGF-ß mutant larvae 24 h after infection with axenic (*Heterorhabditis bacteriophora*, *Hb*) or symbiotic (*Heterorhabditis bacteriophora* + *Photorhabdus luminescens*, *Hb* + *Pl*) nematodes. (**A**) Levels of trehalose in *daw* and *dpp* mutant larvae infected with parasitic nematodes. Trehalose levels are elevated in uninfected *daw* mutant larvae and in *daw* mutants infected with axenic *H. bacteriophora* nematodes compared to their respective background controls (*w*^1118^) (**** *p* < 0.0001). In addition, trehalose levels are decreased in *daw* mutant larvae infected with symbiotic nematodes compared to the uninfected *daw* mutants (*** *p* = 0.0004) and *daw* mutants infected with axenic nematodes (*** *p* = 0.0002). Trehalose content in *dpp* mutant larvae infected with *H. bacteriophora* symbiotic nematodes is significantly higher than in *dpp* mutants infected with axenic nematodes (* *p* = 0.0272). (**B**) Levels of glucose in infected *daw* and *dpp* mutant larvae. Levels of glucose are reduced in uninfected *daw* mutant larvae (** *p* = 0.0017) and those infected with either axenic (* *p* = 0.0386) or symbiotic *H. bacteriophora* (** *p* =0.0036) compared to their background controls (*w*^1118^). (**C**) Levels of glycogen in infected *daw* and *dpp* mutant larvae. There is no significant change in the levels of glycogen in TGF-β mutant larvae upon infection with axenic or symbiotic nematodes. The experiments were performed in biological duplicates and repeated three times (N = 42–54).

Sugar metabolism is essential for maintenance of cellular energy levels in insects. *D. melanogaster* contains glucose and trehalose as circulating sugars, and glycogen as stored sugar [24]. Recent work in *D. melanogaster* larvae has revealed a link between regulation of glycogen metabolism and Activin signaling in response to infection with *H. gerrardi* parasitic nematodes. Specifically, *D. melanogaster* daw mutant larvae infected with *H. gerrardi* nematodes contain elevated glycogen levels compared to uninfected daw mutant larvae [6]. To investigate whether the Activin and BMP signaling branches alter sugar metabolism in *D. melanogaster* in response to *H. bacteriophora* axenic or symbiotic nematodes, we estimated trehalose, glucose, and glycogen levels 24 h post-infection. We found that *daw* mutant larvae contained significantly higher levels of trehalose in the absence of infection (*p* < 0.0001) and in response to infection with axenic nematodes (*p* < 0.0001) compared to their background controls (*w*^111^^8^) (Figure 1A). However, levels of trehalose were significantly lower in *daw* mutant larvae upon infection with symbiotic *H. bacteriophora* compared to uninfected daw mutants (*p* = 0.0004) and larvae infected with axenic nematodes (*p* = 0.0002). In addition, *dpp* mutant larvae infected with symbiotic nematodes contained significantly increased amounts of trehalose compared to those infected with axenic nematodes (*p* = 0.0272). In contrast, *daw* mutant larvae had lower levels of glucose in the absence of infection (*p* = 0.0017) and in response to axenic (*p* = 0.0386) or symbiotic *H. bacteriophora* challenge (*p* = 0.0036) than their background controls (*w*^1118^) (Figure 1B). We did not observe any statistically significant changes in glycogen levels between *daw* and *dpp* mutant larvae infected with axenic or symbiotic nematodes relative to uninfected controls (Figure 1C). These results indicate that the Activin branch of TGF-β signaling in *D. melanogaster* modulates metabolism of circulating sugars in the context of a nematode challenge by increasing the levels of trehalose in larvae responding to symbiotic *H. bacteriophora* infection. 

### 3.2. D. melanogaster dpp Mutants Express dilp3 at Higher Levels in Response to Symbiotic H. bacteriophora Infection

**Figure 2:**
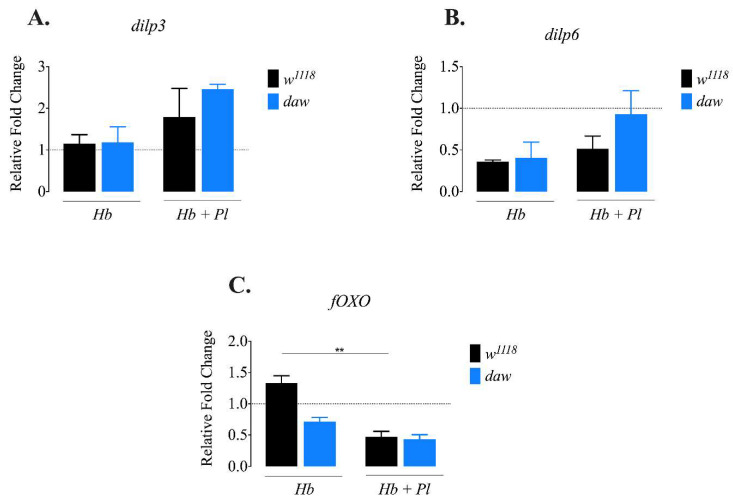
Expression of *dilp3*, *dilp6* and *fOXO* in *Drosophila melanogaster daw* mutant and background control larvae 24 h after infection with axenic (*Heterorhabditis bacteriophora*, *Hb*) or symbiotic (*Heterorhabditis bacteriophora* + *Photorhabdus luminescens*, *Hb* + *Pl*) nematodes. (**A**,**B**) There is no significant change in the expression of *dilp3* and *dilp6* in *daw* mutant larvae after infection with *H. bacteriophora* axenic or symbiotic nematodes compared to their background control (*w*^1118^). (**C**) Expression of *fOXO* is reduced in background control larvae (*w*^1118^) when infected with *H. bacteriophora* symbiotic nematodes compared to uninfected controls (** *p* = 0.0098). Dotted line at 1 indicates normalization of fold change relative to uninfected controls. The experiments were performed in biological duplicates and repeated three times (*N* = 30–42).

**Figure 3:**
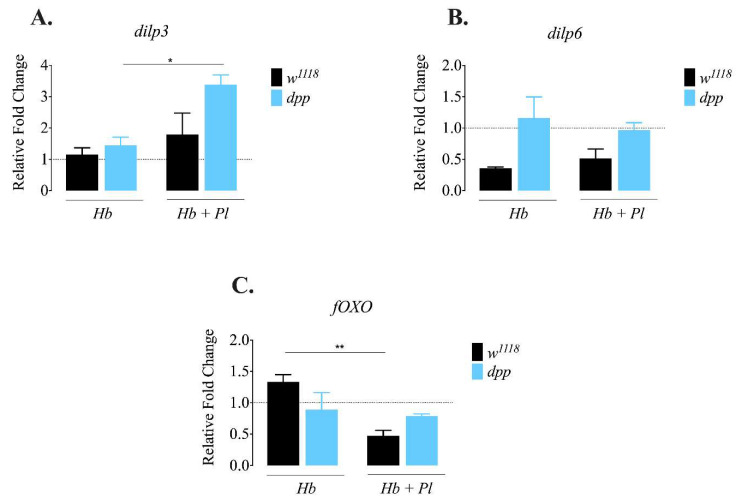
Expression of *dilp3*, *dilp6* and *fOXO* in *Drosophila melanogaster dpp* mutant and background control larvae 24 h after infection with axenic (*Heterorhabditis bacteriophora*, *Hb*) or symbiotic (*Heterorhabditis bacteriophora* + *Photorhabdus luminescens*, *Hb* + *Pl*) nematodes. (**A**) Expression of *dilp3* increases in *dpp* mutant larvae infected with symbiotic *H. bacteriophora* compared to *dpp* mutants infected with axenic *H. bacteriophora* (* *p* = 0.0486). (**B**) There is no significant change in the expression of *dilp6* in *dpp* mutant larvae infected with either axenic or symbiotic nematodes compared to their background control (*w*^1118^). (**C**) Expression of *fOXO* is reduced in background control larvae (*w*^1118^) infected with symbiotic *H. bacteriophora* compared to background control larvae infected with axenic nematodes (** *p* = 0.0098). Dotted line at 1 indicates normalization of fold change relative to uninfected controls. The experiments were performed in biological duplicates and repeated three times (N = 30–42).

The insulin signaling pathway controls essential processes, including the metabolic response in *D. melanogaster* [25]. Drosophila insulin-like peptides (Dilps) maintain the balance between stored and circulating carbohydrates, and regulate lipid metabolism [26,27]. Previous evidence indicates that lack of *dilp3* results in elevated levels of trehalose and glycogen, and lack of *dilp6* increases stored lipid levels in the absence of infection in *D. melanogaster* [28,29]. In addition, it has been shown that insulin signaling interacts with the TGF-ß signaling in the absence of infection. More precisely, the Activin pathway interacts with insulin signaling through Daw by positively regulating the release of Dilps in *D. melanogaster* larvae [30]. To investigate a potential link between insulin signaling and TGF-β signaling in response to parasitic nematode infection, we used qRT-PCR and gene-specific primers to determine the transcript levels of *dilp3*, *dilp6*, and transcription factor *fOXO* in *daw* and *dpp* mutant larvae 24 h after infection with *H. bacteriophora* axenic or symbiotic nematodes. We found no statistically significant differences in *dilp3* and *dilp6* levels in *daw* mutant larvae after infection with *H. bacteriophora* axenic or symbiotic nematodes compared to their background controls (*w*^1118^) (Figure 2A,B). However, expression of *fOXO* was reduced in background control larvae (*w*^1118^) infected with *H. bacteriophora* symbiotic nematodes compared to uninfected controls (Figure 2C; *p* = 0.0098). We further noticed that the expression of *dilp3* was higher in *dpp* mutant larvae infected with symbiotic *H. bacteriophora* compared to *dpp* mutants infected with axenic nematodes (Figure 3A; *p* = 0.0486). We observed no statistically significant differences in the transcript levels of *dilp6* in *dpp* mutant larvae infected with either axenic or symbiotic nematodes compared to their background controls (*w*^1118^) (Figure 3B). These results imply that the activity of BMP branch of the TGF-ß signaling pathway in *D. melanogaster* larvae might be involved in suppressing *dilp3* expression upon infection with *H. bacteriophora* symbiotic nematodes.

### 3.3. The BMP Branch Regulates Metabolism of Stored Fats in D. melanogaster Larvae in the Context of H. bacteriophora Infection

**Figure 4:**
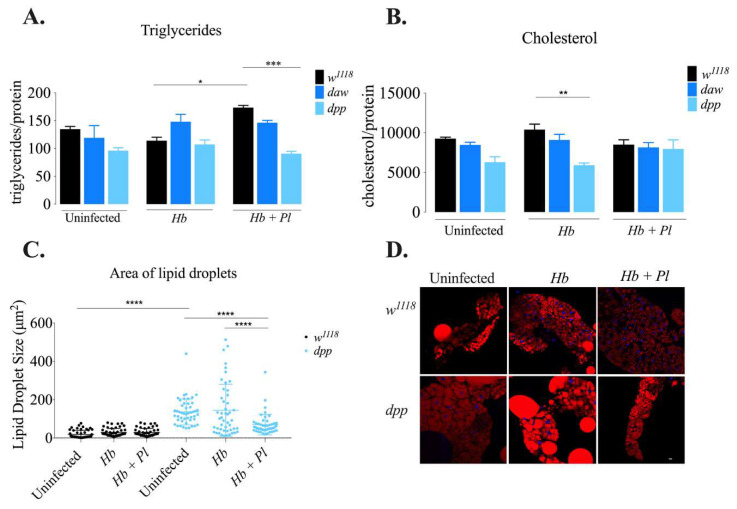
Lipid metabolism in *Drosophila melanogaster* TGF-β mutant larvae 24 h after infection with axenic (*Heterorhabditis bacteriophora*, *Hb*) or symbiotic (*Heterorhabditis bacteriophora* + *Photorhabdus luminescens*, *Hb* + *Pl*) nematodes. (**A**) Triglyceride levels in TGF-ß mutant larvae infected with parasitic nematodes. Triglyceride content in *dpp* mutant larvae significantly decreases after infection with *H. bacteriophora* symbiotic nematodes compared to background control (*w*^1118^) larvae (*** *p* = 0.0004). In addition, following symbiotic *H. bacteriophora* infection, level of triglycerides increase in background control (*w*^1118^) larvae compared to larvae infected with axenic nematodes (* *p* = 0.0164). (**B**) Cholesterol levels in TGF-ß mutant larvae infected with parasitic nematodes. Cholesterol content is reduced in *dpp* mutants infected with axenic *H. bacteriophora* compared to background control larvae (** *p* = 0.0038). (**C**) Size of lipid droplets in the fat body of *D. melanogaster dpp* mutant larvae 24 h after infection with *H. bacteriophora* axenic or symbiotic nematodes (**** *p* < 0.0001). (**D**) Confocal microscopy images of lipid droplets stained with Nile Red (red) and DAPI (blue) in the fat body of *H. bacteriophora*-infected or uninfected *D. melanogaster dpp* mutant larvae and their background controls (*w*^1118^). Scale bar is 10 microns. Triglyceride and cholesterol quantification experiments were performed in biological duplicates and repeated three times (*N* = 42–54). Lipid droplet staining was performed by measuring the four largest lipid droplets per cell from four fat body cells.

Lipid metabolism has a central role in maintaining energy homeostasis in insects. In *D. melanogaster*, stored fats such as triacylglycerol (TAG) and cholesterol mostly accumulate in the fat body, the functional equivalent of the mammalian liver [31]. Previous work has demonstrated that in the absence of infection, TGF-β signaling activity in hepatocytes contributes to lipid accumulation in mice [32]. In addition, the extracellular ligand Gbb in the BMP branch is required in the fat body of uninfected *D. melanogaster* larvae to modulate lipid metabolism [33]. To investigate the interaction between the Activin or the BMP branch of the TGF-β signaling and the metabolism of stored lipids in the context of parasitic nematode infection, we determined the levels of TAG and free cholesterol in *daw* and *dpp* loss-of-function mutant larvae 24 h after infection with axenic or symbiotic *H. bacteriophora*. We found that TAG levels in *dpp* mutant larvae significantly decreased after infection with symbiotic *H. bacteriophora* compared to background control larvae (*w*^1118^) (Figure 4A; *** *p* = 0.0004). On the contrary, cholesterol content was reduced in *dpp* mutant larvae in response to infection with axenic nematodes compared to background controls (Figure 4B; *p* = 0.0038). However, we found no statistically significant differences in TAG and cholesterol levels in *daw* mutant larvae after infection with *H. bacteriophora* axenic or symbiotic nematodes compared to their control individuals (Figure 4A,B). 

Lipid droplets are specialized energy storage organelles that play an essential role in lipid metabolism and cellular homeostasis in *D. melanogaster* [34]. Recent work has shown that the size of lipid droplets in *D. melanogaster dpp* mutant larvae infected with *H. gerrardi* nematodes increases compared to uninfected larvae [6]. To further assess the interaction between the BMP branch and lipid metabolism, we stained fat body lipid droplets with Nile red and DAPI, and measured lipid droplet size in *dpp* loss-of-function mutant larvae 24 h after infection with *H. bacteriophora* axenic or symbiotic nematodes (Figure 4C,D). We found that the size of fat body lipid droplets significantly decreased in *dpp* mutant larvae after infection with *H. bacteriophora* symbiotic nematodes compared to uninfected larvae (Figure 4C; *p* < 0.0001). In addition, *dpp* mutant larvae infected with symbiotic nematodes contained smaller lipid droplets than larvae infected with axenic nematodes (*p* < 0.0001). These findings suggest that the BMP/Dpp branch of the TGF-β signaling pathway in *D. melanogaster* larvae regulates the metabolism of TAG in response to *H. bacteriophora* symbiotic nematodes, and cholesterol in response to axenic nematodes. It also modulates fat body lipid droplet size upon infection with symbiotic nematodes. 

### 3.4. ATP Levels in D. melanogaster daw and dpp Mutant Larvae Are Unaffected in Response to H. bacteriophora Infection 

**Figure 5:**
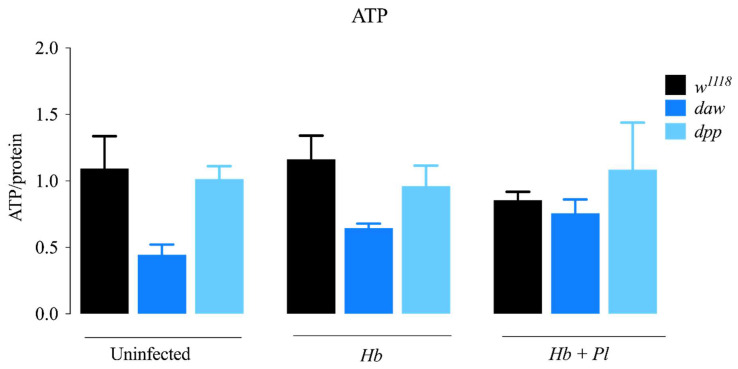
Levels of ATP in *Drosophila melanogaster* TGF-ß mutant larvae 24 h after infection with axenic (*Heterorhabditis bacteriophora*, *Hb*) or symbiotic (*Heterorhabditis bacteriophora* + *Photorhabdus luminescens*, *Hb* + *Pl*) nematodes. There is no significant change in ATP levels between either *daw* or *dpp* TGF-ß mutant and its background control (*w*^1118^). The experiment was performed in biological duplicates and repeated three times (*N* = 42–54).

Adenosine triphosphate (ATP) is found in all living cells and is responsible for energy storage and transfer. Quantification of ATP provides direct information on cellular energy levels which is associated with the metabolic state of the organism [23]. To understand whether the Activin and the BMP branches of the TGF-β signaling pathway modulate cellular energy levels in *D. melanogaster* responding to nematode parasites containing or lacking their symbiotic bacteria, we infected larvae with *H. bacteriophora* symbiotic or axenic nematodes and then measured the levels of ATP using a bioluminescence assay. We found that following nematode infection, there were no significant changes in ATP levels between either *daw* or *dpp* TGF-β mutants compared to their background controls (*w*^1118^) (Figure 5). These results imply that the Activin and BMP TGF-β signaling branches do not modulate ATP levels in *D. melanogaster* larvae during infection with *H. bacteriophora* nematodes.

## 4. Discussion

Pathogens and parasites improve and/or extend the duration of their infection capacity and transmissibility by inducing alterations in host metabolic processes [35,36]. Recent evidence has shown that the components of the Activin and the BMP branches of the TGF-β signaling pathway contribute to the metabolic response of *D. melanogaster* larvae against infection with *H. gerrardi* nematodes, which naturally associate in a mutualistic symbiosis relationship with the bacteria *P. asymbiotica* [6]. However, whether the interaction between TGF-ß signaling activity and the *D. melanogaster* anti-nematode metabolic response is regulated by the nematodes alone without any potential influence from their natural found bacterial symbionts is not thoroughly understood. Therefore, we investigated the interaction between the Activin and the BMP signaling activity with the *D. melanogaster* metabolic response against infection with axenic or symbiotic *H. bacteriophora* nematodes. Here we report that the Activin signaling interacts with sugar metabolism while the BMP signaling mediates metabolism of lipids in *D. melanogaster* during the response to infection with axenic or symbiotic *H. bacteriophora* parasitic nematodes.

Previous studies have highlighted the importance of the Activin and BMP signaling branches in regulating *D. melanogaster* sugar metabolism in the absence of infection. The extracellular Activin branch ligand Daw regulates metabolic processes in *D. melanogaster* larvae in a positive manner. In addition, loss-of-function mutation in *daw* is associated with disruption in sugar homeostasis [30]. Similarly, loss-of-function mutation in the gene coding for the extracellular BMP branch ligand Gbb results in decreased glucose and trehalose levels [33]. In our study, we have also observed a link between Activin signaling activity and *D. melanogaster* sugar metabolism, however in the context of parasitic nematode infection. We have further demonstrated that *daw* deficient larvae contain elevated levels of trehalose in response to infection with axenic *H. bacteriophora* compared to their background control (*w*^1118^). In contrast, levels of trehalose were significantly decreased in *daw* mutants upon infection with symbiotic nematodes compared to uninfected *daw* mutants or larvae infected with axenic nematodes. This implies that the *H. bacteriophora*-*P. luminescens* nematode-bacteria complex can suppress the levels of trehalose in the absence of a functional Activin signaling branch. Of note, our previous results in *D. melanogaster* adults have revealed that injection of either *P. luminescens* or *P. asymbiotica* pathogenic bacteria into the hemolymph of wild type flies leads to a decrease in the levels of trehalose compared to uninfected individuals [37]. Taken together, our current findings suggest that the Activin branch of the TGF-β signaling modulates trehalose metabolism in *D. melanogaster* larvae upon symbiotic *H. bacteriophora* nematode infection.

Lipid droplets consisting of TAGs and cholesteryl esters are considered as essential regulators of lipid metabolism [38]. Our previous studies have demonstrated that the change in lipid droplet status in the *D. melanogaster* larval fat body depends on the type of parasitic nematode infection, where *Steinernema carpocapsae* infection increases their size [39], but *H. gerrardi* infection results in a decrease [6]. In addition, no alterations in TAG levels were found in these studies. Similarly, in our current study we did not find any variation in TAG amounts and in addition no change in lipid droplet size was observed in the background control larvae responding to axenic or symbiotic *H. bacteriophora* infection compared to uninfected larvae. These differences might imply parasite-specific regulation of the host metabolic response. In addition, the Activin branch has a critical role in the regulation of lipid metabolism. The extracellular Activin signaling ligand Babo reduces TAG levels in the *D. melanogaster* larval fat body and promotes mobilization of lipids [40]. Our previous work has shown that the Activin and BMP branches participate in the regulation of lipid metabolism in *D. melanogaster* during response to parasitic nematode infection. More precisely, we have shown that lipid droplets in both *daw* and *dpp* mutant larvae increase in size upon infection with *H. gerrardi* nematodes compared to uninfected larvae [6]. Here we report that lipid droplet size decreases in *dpp* deficient larvae against infection with symbiotic *H. bacteriophora* nematodes compared to uninfected mutants. Both *H. gerrardi* (containing *P. asymbiotica* bacteria) and *H. bacteriophora* (containing *P. luminescens* bacteria) nematodes belong to the *Heterorhabditidae* family of parasitic nematodes, but they obviously interact differently with the host lipid droplets during infection. Hence, it is possible that variation in these effects might be due to differences in the biology of the symbiotic bacteria that inhabit the intestine of *H. gerrardi* and *H. bacteriophora* nematodes [41]. Interestingly, investigating the lipid content in BMP deficient larvae challenged with *H. bacteriophora* revealed a decrease in TAG levels in *dpp* mutants upon symbiotic nematode infection and a decrease in cholesterol levels upon axenic nematode infection compared to their background controls. Our findings suggest that the BMP signaling branch modulates lipid metabolism through regulating TAG and cholesterol levels, which is reflected by changes in the size of fat body lipid droplets during *H. bacteriophora* infection.

In summary, our study reports that the Activin signaling in *D. melanogaster* modulates sugar metabolism by increasing the levels of trehalose in response to symbiotic *H. bacteriophora* infection. In addition, the BMP signaling interacts with the metabolism of TAGs against infection with *H. bacteriophora* symbiotic nematodes, and the metabolism of cholesterol against infection with axenic nematodes. The BMP signaling also contributes to the regulation of fat body lipid droplets in larvae responding to *H. bacteriophora* nematode infection. Our observation that TGF-β signaling activity in *D. melanogaster* can modify host metabolic processes during a response to infection with parasitic nematodes provides a new basis for the prevention and control of metabolic disorders associated with parasitic diseases in vertebrates, perhaps including humans. Knowledge of this information might contribute to the identification of alternative possibilities for adopting novel anti-parasitic treatment strategies in the future.

## Figures and Tables

**Table 1:** Primers and their sequences used in quantitative RT-PCR experiments.

Gene	Accession No	Primer (5′–3′)	Sequence	Tm (°C)
***RpL32***	CG7939	Forward	GATGACCATCCGCCCAGCA	60
		Reverse	CGGACCGACAGCTGCTTGGC	
***Dilp6***	CG14049	Forward	ATATGCGTAAGCGGAACGGT	57
		Reverse	GCAAGAGCTCCCTGTAGGTG	
***Dilp3***	CG14167	Forward	AGAGAACTTTGGACCCCGTGAA	59
		Reverse	TGAACCGAACTATCACTCAACAGTCT	
***fOXO***	CG3143	Forward	AGGCGCAGCCGATAGACGAATTTA	60
		Reverse	TGCTGTTGACCAGGTTCGTGTTGA	
